# Macau parents’ perceptions of underage children’s gambling involvement

**DOI:** 10.1186/s40405-017-0021-8

**Published:** 2017-03-03

**Authors:** Ernest Moon Tong So, Yorky Mei Po Lao, Irene Lai Kuen Wong

**Affiliations:** 1 Department of Sociology, University of Hong Kong, Pokfulam Road, Pokfulam, Hong Kong SAR, China; 2 Macao Public Security Police Force, Macao, China; 3 Department of Applied Social Sciences, Hong Kong Polytechnic University, Hung Hom, Kowloon, Hong Kong

**Keywords:** Children gambling, Parent gambling, Macau survey, Prevention

## Abstract

**Objectives:**

The study examined Macau parents’ perceptions of underage children’s gambling involvement, and parents’ attitudes towards help seeking if their children had a gambling problem. The parents’ gambling behavior in the past year was also investigated.

**Methods:**

This is a parent survey using a self-administered questionnaire. A convenience sample of 311 Macau parents (106 fathers and 205 mothers) with underage children aged 3–17 years was recruited. The response rate is 77.8%. The participants were asked if they had ever approved or taught their underage children to gamble, and how did they award their children when they won in gambling games. The parents were also asked if they had gambled in the previous 12 months, and their gambling behavior was assessed by the Chinese Problem Gambling Severity Index (CPGSI).

**Results:**

Half of the parents surveyed (52%) did not approve underage gambling but 81% taught their underage children to play different gambling games. Children were awarded with money (55%), praises (17.5%), toys (15%) and food (12.5%) when they won in games. One-fifth (20.6%) were distressed with their children’s gambling problem. Many (68.8%) were willing to seek help to cope with children’s gambling problems. Only 21.2% (n = 66) of the parents reported gambling in the past year. Using the CPGSI, 4.5% of these gamblers could be identified as problem gamblers, and 16.7% were moderate-risk gamblers.

**Conclusion:**

The study results indicate parent education should be included in prevention of underage gambling.

## Background

Underage gambling is common in different parts of the world. There has been a lot of evidence suggesting at least 60% of underage youths and children had gambled in the previous year (Adlaf and Ialomiteanu 1999; Adlaf et al. 2006; Delfabbro et al. 2005; Derevensky et al. 1996; Felsher et al. 2003; Gupta and Derevensky 1997; Ladouceur et al. 1994; Ladouceur and Mireault 1988; Wong 2010a, b). As high as 90% of adolescent students reported that their parents knew they had gambled, and 80% thought their parents did not disapprove (Ladouceur and Mireault 1988).

Many of these young gamblers made their first bet at an early age often in a family context. Plenty of research data verify youth problem gambling correlates with early gambling initiation, often before the age of 10 years (e.g. Dickson et al. 2004; Jacobs 2000; Winters et al. 2002; Wong 2010a, b; Wynne et al. 1996). Retrospective reports from adult problem gamblers recruited from Gamblers Anonymous (1997) also confirmed early gambling could be harmful. Many of these gamblers began gambling early in life. In Macau gambling under 18 years is unlawful but minors under 21 years are not permitted to enter and play games at any Macau casino. Illegal gambling among the Macau underage is liable to fines and penalties.

Who introduce these young people to gambling activities? Chinese parents, elder siblings and relatives often teach young children and teenagers to play various gambling games in family gatherings, especially at festival celebration events such as in the Chinese New Year celebration activities. In a survey in which 1001 Chinese underage adolescents were recruited, Wong (2010a) noted that 60% reported gambling in the preceding year. Many young gamblers were first introduced to gambling in a family context by their parents (40.5%) and family members such as older siblings (22.3%). They were also assisted by their parents (44.7%) to wager at both legal and illegal outlets. More than half (55.2%) claimed that their parents bought lottery tickets for them, and 50.8% were helped to bet on horse races and soccer matches (28%).

Western studies also verify that children gamble with parents and family is a common occurrence (Gupta and Derevensky 1997; Moore and Ohtsuka 1997). As high as 80% of Canadian children (Gupta and Derevensky 1997) had gambled with family members such as parents (40%), siblings (53%) and relatives (46%). Lotteries, scratch cards, card games and sports betting are popular family gambling games. Many parents bought lottery tickets and scratch cards for their children (Ladouceur et al. 1998; Ladouceur and Mireault 1988; Wood and Griffiths 1998). Gambling on lotteries is often perceived by adults as a harmless activity (Winters et al. 1995).

Parental influence has significant impact on children’s gambling involvement and problem gambling. Felsher et al. (2003) pointed out that tacit approval was given when parents purchased lottery tickets for their children. Such approval would likely produce more serious children gambling involvement or support already problematic gambling. Parents may overlook the fact that they are the role models for their children. Children often follow parents’ gambling behavior, develop gambling attitudes and cognitions similar to those of their parents (Oei and Raylu 2004). If parents teach or allow their children to gamble with them at home, such parental behavior often implies gambling is accepted as a family activity.

Children of gamblers and problem gamblers are at increased risk of having gambling problems (Gupta and Derevensky 1998; Felsher et al. 2003; Hsu et al. 2014; McComb and Sabiston 2010; Vachon et al. 2004; Vitaro and Wanner 2011; Wickwire et al. 2007; Wong 2010a). Problem gambling is characterized by dependence, loss of control and harmful consequences (American Psychiatric Association 2013). Wickwire et al. (2007) reported that teens with a gambling parent were 2.8 times more likely to report at-risk or problem gambling. Hsu et al. (2014) also found that Chinese adolescent students distressed by parental problem gambling were 1.6 times more likely to develop pathological gambling, and 2.7 times more likely to be at-risk gamblers, although only 16.7% of 1095 high school students surveyed disclosed parental problem gambling. One-fifth (20.2%) reported gambling with one of their parents.

Many studies have documented parental influence on children’s gambling behaviour by asking the children. Few researches have investigated parents’ perceptions and attitudes towards children’s gambling behaviour (Campbell et al. 2011). In this study, Chinese parents residing in Macau were the target survey participants. To our knowledge, parent studies on this topic do not exist in Macau. This study was the first survey launched to provide empirical evidence on this under examined topic. We aimed to explore Macau Chinese parents’ perceptions (i.e. parental views) of underage children’s gambling behaviour, and parents’ readiness to seek professional help to cope with children’s gambling problem. Parents’ opinions on prevention programs organized for children and adolescents were collected as well. We would also like to explore if parent gamblers were more likely to approve underage children to gamble than non-gamblers. Hence, parents’ gambling involvement in the preceding year was investigated. The study findings throw light on public and parent education programs.

## Methods

This is a parent survey using a standardized self-administered questionnaire. Using the convenience sampling strategy, a total of 400 questionnaires were distributed to the potential survey participants recruited from parent services agencies and parental networks randomly selected from a directory of service organizations and networks developed by the research team. A trained Macau researcher sent out and collected the completed questionnaires after program termination at the parents’ organizations. It only took 15 min to fill the anonymous questionnaire. A total of 311 properly filled and valid questionnaires were returned, generating a response rate of 77.8%.

### Participants

All the 311 survey participants were Chinese parents residing in Macau. There were 205 mothers (65.9%) and 106 (34.1%) fathers. A quarter of the parents (25.7%) aged above 51 years, 20.5% (n = 63) aged 41–45 years, 19.9% (n = 61) aged 46–50 years, 19.2% (n = 59) aged below 36 years, and 14.7% (n = 45) were within the age bracket of 36–40 years.

All the parents had underage children aged 3–17 years. More than half (58%) had two children, 33% had one child, 10% had three children, and 2% had four children. Most completed secondary school education (43%), 23% attended primary schools, 17% had a university degree, and 15% finished post-secondary education. Only 2% did not receive any formal education.

Ninteen percent of the parents were civil servants, 16% were house-wives, 12% were casino croupiers, 10% were professional and managerial employees, 8% were employees of the tourism industry, and the rest came from different vocations (35%) without providing clear information about their jobs.

### Measures

A Chinese self-administered questionnaire was filled by the survey participants with informed consent and assurance of anonymity. Participation was voluntary. There are four components in the questionnaire:

- The parents answered questions on socio-demographic background (e.g. sex, age, education level and employment status),
- Questions were designed to collect information on parents’ attitudes and perceptions of underage children’s gambling involvement. For example, parents were asked if they had ever approved or introduced their children to gambling games. How would they award their children when they won in gambling activities involved betting with money? Parents were also asked to give their views freely on underage gambling by providing descriptions (e.g. underage gambling was a game, a memory enhancer, a children recreation).
- Questions were developed to gather data on underage children’s gambling behavior. For example, parents were asked if their children had ever requested to gamble with adult family members, and if their children had ever been invited to gamble by adult family members and relatives within and outside the family environment. How old were their children when they received the first gambling invitation? Have their children ever had a gambling problem and from whom they would seek help to cope with children’s gambling problem?
- Parents were asked if they had gambled in the past year, and had gambled at home with their underage children around since they were born. We wonder if more gambling parents would approve underage children to gamble than the non-gamblers.
- Parents’ past year gambling behavior was assessed by an adapted Chinese 9-item Problem Gambling Severity Index (CPGSI) (Loo et al. 2011) to make it culturally appropriate for the Chinese parents in Macau. The English PGSI was developed by Ferris and Wynne (2001) which has been translated into many languages including the Chinese language. The Chinese language translation has not caused differential item functioning (Sharp et al. 2012). It only takes a few minutes to fill the CPGSI which has been verified to be reliable (Cronbach’s alpha = 0.77) and valid. The CPGSI is a unidimensional measure with good concurrent, discriminant and predictive validity (Loo et al. 2011).
  Responses are rated on a 4-point Likert scale (0 = never, 1 = sometimes, 2 = most of the time, 3 = almost always). Scores range from 0 to 27. Non-problem gamblers score 0 on the screen. Low-risk gambling (a score of 1–2) has no or few gambling harms. Moderate-risk gambling (a score of 3–7) results in some harmful consequences. Problem gambling (a score exceeds 7) suggests negative consequences and a possible loss of control.
  (d) In order to examine the participants’ opinions on prevention of underage gambling problems, parents were asked if they felt being responsible for teaching children about potential gambling harms, and who should also be sharing this education responsibility. Lastly, the parents were asked if they were aware of any measure or program aiming at preventing problem gambling among children and adolescents in the previous year.

### Statistical analysis

Data collected were analyzed by using the SPSS (version 19). Descriptive statistical tests were used to run frequencies, percentages, and Chi squares. Attempts were also made to assess the association between parental attitudes towards children gambling and parental gambling problems. Results are noted significant at *p* < 0.05.

## Results

### Parents taught their children to gamble

Majority of the participants (n = 252, 81%) taught their underage children to play various gambling games, including mahjong (41.3%), card games (36.1%), Mark 6 lottery (22.6%), dice games (11.5%), soccer betting (6.7%) and horse racing (2.4%). Among 40 parents who awarded their children when they won in gambling games, money (55%), verbal recognition and praises (17.5%), toys (15%) and food (12.5%) were provided.

### Parental approval for underage gambling

Although most parents (81%) taught their children to play different gambling games, many (n = 162, 52%) reported that they would not approve underage gambling, only 32% would allow their children to gamble with small wagers, and 15.8% did not care if their children gamble or not before they had reached the legal age of gambling in Macau. The legal age for casino gambling in Macau is 21 years, and teens of 18 years are allowed to purchase lottery tickets.

### Parental perceptions of children gambling

Among 290 parents who answered the question on parental perceptions of children gambling, 31% (n = 90) accepted the activity as a children recreation, 27.9% (n = 81) viewed gambling as a game, 6.6% (n = 19) regarded it as one type of family activities, and 4.3% (n = 12) viewed it as a memory enhancer for children. Only 30.2% (n = 88) considered gambling could be dangerous and harmful to their children.

### Family members gambled with underage children

Among 288 parents who answered the question on whether their children had been invited to gamble by family members and relatives, 16% (n = 46) reported that their children did so in their house (50%) and in their relatives’ house (43.4%), or even in a restaurant (6.5%) or in a park (6.5%).

These family members were grandparents (50%) and elder siblings (50%). Relatives were uncles and aunts. The gambling activities were mahjong (37.0%), card games (34.8%), Mark Six lotteries (10.7%), dice games (8.7%), soccer betting (6.5%), and wagering on horse and grey hound races (4.3%).

### Children requested to gamble with adults

Apart from being invited to play with adults, at least 9.6% (n = 30) of the parents revealed that their children had requested to gamble with adult family members, 76.2% denied knowing such requests, and 14.2% were not sure if such requests had ever been made by their children.

Only 10% of the survey participants (n = 31) reported that they knew their underage children had gambled with people other than their parents and family members. Majority (90%) believed their children had never done so.

### Age at which children were invited to gamble

Many children were invited to gamble with money by adult family members and relatives at least once a year (61%) in Chinese festivals, quarterly (23%) or monthly (16%). Many parents noted that the first gambling invitation was made when their children were between 12 and 14 years (43.1%), while 27.3% reported a younger age of 6–11 years, and 2.3% disclosed 2–5 years. Only 27.3% reported an older age range of 15–17 years.

### Parental concern about children’s gambling problems

At least 20.6% (n = 64) of the parents were distressed with children’s gambling behavior, 8.0% (n = 25) were not sure if their children had a gambling problem or not. Majority (n = 222, 71.4%) believed their children did not have a gambling problem when the study was conducted.

### Parents’ readiness to seek help to cope with children problem gambling

Many parents (n = 214, 68.8%) reported that they would seek both formal and informal help if their children had a gambling problem. Formal help providers included school teachers (n = 81, 37.9%), social workers (n = 69, 32.2%) and gambling counselors (n = 37, 17.3%). Informal help could be sought from relatives (n = 27, 12.6%).

### Parents’ gambling involvement

Among 289 parents who answered the question on parents’ gambling behavior, more than one-quarter (n = 74, 25.6%) had gambled at home since their children were born. They often played mahjong (n = 35, 47%), bought Mark 6 lottery tickets (n = 15, 20.5%), and played card games (n = 12, 16.2%) in the presence of their underage children. Some staked on horse racing (n = 12, 16.3%), Internet gambling (n = 12, 16.3%) and soccer betting (n = 12, 16.3%).

### Problem gambling among the parents

Only 21.2% (n = 66) of the 311 participants reported gambling in the past year (Table 1). Using the PGSI (Ferris and Wynne 2001), 68.2% of the parents (n = 45) were non-problem gamblers, 10.6% (n = 7) were low-risk gamblers, 16.7% (n = 11) were moderate-risk gamblers, and 4.5% (n = 3) could be categorized as problem gamblers.

**Table 1:** Classification of gambling behavior using the CPGSI (n = 66)

Gambling behavior	Gender		Total
	Males (n and %)	Females (n and %)	
Non-problem gambling	12	33	45
	57.1%	73.3%	68.2%
Low-risk gambling	5	2	7
	23.8%	4.4%	10.6%
Moderate-risk gambling	4	7	11
	19.0%	15.6%	16.7%
Problem gambling	0	3	3
	0.0%	6.7%	4.5%
Total	21	45	66
	100.0%	100.0%	100.0%

### Parents’ opinions on prevention of children problem gambling

Among 289 parents who provided opinions on prevention of children problem gambling, 24.3% (n = 70) accepted that parents should be responsible for teaching their children about potential gambling harms, while 23.2% (n = 67), 16.8% (n = 49), 18.1% (n = 52) and 17.6% (n = 51) of the participants suggested that the education responsibility should be shared by school teachers, social workers, the Macau government and the social media respectively.

During the past 12 months, 79.1% (n = 230) of 291 parents reported that they were aware of at least one of the measures or programs implemented to prevent children and adolescent problem gambling activities, but 20.9% (N = 61) did not. The prevention initiatives included radio and television warning messages (59.5%), information pamphlets (41.2%), educational movies (39.9%), programs organized by gambling treatment centres (34.7%), school and community exhibitions (20.3%).

### Approval of children gambling among problematic gamblers

Problematic gamblers (i.e. the cases of problem gamblers and moderate-risk gamblers were combined) were significantly more likely to approve underage children to gamble than the non-problematic gamblers ($\chi^{2}$(3) = 11.0, *p* < 0.05). However, no significant difference was found between the gamblers and the non-gamblers ($\chi^{2}$(1) = 0.8, *p* > 0.05). The gambling parents as a whole were not significantly more likely to approve underage children gambling than the non-gamblers.

## Discussion

Consistent with previous research results, this study confirmed parental gambling involvement with children was popular (Campbell et al. 2011; Gupta and Derevensky 1997; Moore and Ohtsuka 1997). Majority of Macau parents surveyed (81%) reported gambling with their underage children. Children also gambled with adult family members and relatives. They were even awarded with money, praises, toys and food when they won in gambling games. We need family-based public education and prevention programs which would include parents and family members such as elder siblings, grand-parents and relatives.

We argue that it is necessary to improve parent education because of the following reasons: First, although a high proportion of the parents (79.1%) were aware of at least one measure or program implemented to prevent underage gambling in the previous year, and many (68.9%) reported their willingness to seek help if their children had a gambling problem, only 24.3% accepted that parents should be responsible for teaching their children the potential gambling harms. More than 75% of the parents suggested that the education responsibility should be shared by other stakeholders (e.g. school teachers: 23.2%, Macau government: 18.1%; social media: 17.6%; social workers: 16.8%). In short, majority (75.7%) did not recognize they had a role to play in providing education to their children to prevent gambling problems.

Furthermore, similar to past research findings (e.g. Campbell et al. 2011), many Macau parents did not perceive children gambling as a potentially harmful or serious issue. Only 30.2% of the Macau parents recognized underage children gambling could be harmful. More than 70% perceived children gambling from a positive perspective including accepting the activity as a children recreation (31%), a game (27.9%), a family activity (6.6%) or even a memory enhancer (4.3%). Above all, 81% of the parents taught their children to play different gambling games with low awareness of potential damages. All these results indicate it is necessary to improve parent education programs by promoting parental awareness of the potential risks and harms associated with children gambling. Parents should also have information on the negative impact of parental gambling on their children. They should be helped to recognize and fulfill their role in preventing children gambling problems.

Secondary prevention should target the problematic gambling parents (i.e. the moderate-risk and problem gamblers), especially the mothers because more mothers than fathers (10 vs. 4) exhibited signs of problematic gambling. The study indicates these parents are more likely to approve underage children to gamble than the non-problematic gamblers. Secondary prevention and parent education should target these parents especially the mothers. Prevention may focus on changing the parents’ attitude, and discourage them to approve underage gambling within and outside the family environment. Parental awareness of the potential negative impact of their gambling behavior on their children should also be increased. Abundant research evidence confirms children of problematic gamblers are more vulnerable to gambling problems (e.g. Felsher et al. 2003; Hsu et al. 2014; McComb and Sabiston 2010; Vachon et al. 2004; Vitaro and Wanner 2011; Wickwire et al. 2007; Wong 2010a). Information on helpline and treatment services should be provided to these parents who may need professional help.

In addition to promoting help seeking among problem gambling parents, it is necessary to further encourage Macau parents to seek professional help if their children are distressed with gambling problems. Only 68.9% of the parents surveyed were willing to seek help to cope with children’s gambling problems. Why were the other 30% reluctant to seek help? Further investigation is needed to identify the barriers to seeking professional help, and more effective and vigorous public promotion is required to encourage both parents and children to seek help.

The study indicates more adolescent children (70.4%) were invited to gamble than those under 12 years old (29.6%). We argue that the underage should not be invited to gamble regardless of their age. Early school-based education programs should be implemented by teachers and school counselors to teach these young people how and why to decline adults’ gambling invitations. Ideally early education should be launched in the primary schools before the children have been invited or tempted to gamble. School-based education should continue throughout the pre-adolescence and adolescence years.

Many Macau parents expected all the key stakeholders (e.g. the Macau government, the teachers, the gambling operators, the social workers and the mass media) have to join hands to prevent underage gambling and problem gambling. The government has to work out long term policy for coordinating and enhancing joint efforts and ventures to be developed by these stakeholders. Parents themselves should also play an active role in preventive programs and family education.

Interestingly, 79% of the Macau parents surveyed were aware of at least one of the preventive measures on children gambling but 81% taught their underage children to gamble. We wonder how useful and effective these preventive measures and programs were. Campbell et al. (2011) also reported that only 35% of the Canadian parents who were aware of youth gambling programs described the available information on gambling as good or excellent. It is necessary to conduct systematic evaluation on the effectiveness of children and youth gambling prevention programs in order to seek improvement.

## Conclusion

To conclude, this study provides evidence-based information about Macau Chinese parents’ perceptions of underage gambling. The information is useful for designing parent awareness and prevention programs. More research is needed to further increase our understanding of parental attitude towards children gambling because little research on this important but under-examined topic has been conducted in Macau.

This is an exploratory study with several limitations. First, a small sample of parents was recruited by convenience sampling strategy. Hence, the study results may not have a high level of generalizability. Future research should include larger samples of parents and other family members if resources are available. Second, we did not make any attempt to include children samples to match with or compare with the parents’ data. It would be interesting to include underage children samples in future studies. Third, improvement in measures will also be beneficial in future research. We failed to find validated instruments to measure parental perceptions of underage children in this study. Therefore, we resorted to designing simple questions to examine parental perceptions. We hope interested researchers would assist in constructing reliable and valid instruments to investigate this topic. In short, there is much more room for improvement in future research as far as the research sampling strategies, the sample size and research instruments are concerned.
